# Risk analysis to reduce the appearance of antibiotic-resistant microorganisms in drinking water, dust and manure of broiler farms through adjustments of management measures

**DOI:** 10.1016/j.psj.2026.106411

**Published:** 2026-01-08

**Authors:** Theresa M Liegsalz, Mykhailo Savin, Céline Heinemann, Julia Steinhoff-Wagner

**Affiliations:** a Professorship of Animal Nutrition and Metabolism, Technical University of Munich, Freising, Germany; b Institute for Hygiene and Public Health, University Hospital Bonn, Bonn, Germany; c Institute of Animal Science, University of Bonn, Bonn, Germany; d HEF World Agricultural System Center, Technical University of Munich, Freising, Germany

**Keywords:** Chicken, Health, Pathogen, Antibiotic-resistant microorganism, Environment

## Abstract

The treatment with antibiotics during the fattening of broilers has stagnated in Germany over the last years, while the national aim is to further reduce the use of antibiotics in human and veterinary medicine due to the occurrence of antibiotic-resistances in microorganisms. Therefore, the aim of this study was to identify relevant management measures in broiler farms which improve the health status of broilers and could reduce the occurrence of antibiotic-resistant, opportunistic pathogens in the broilers’ environment. A survey was conducted querying existing management measures based on seven different subsections (general operating data, fattening data, management technical data, housing climate, feeding system, health information, cleaning system). Additionally, samples of water, dust and manure were taken in the last week of an average fattening period, and analysed for the occurrence of *Staphylococci, Staphylococcus aureus (****S. aureus****)*, methicillin-resistant *S. aureus* (**MRSA**) and species with resistance to extended-spectrum beta-lactams. The management measures of the participating farms (*n* = 14) were heterogeneous, with significant differences comparing conventional and organic operating farms, e.g., the number of animals per flock or the duration of the fattening period (in days). Evaluating the predictors which reduce or increase the risk of antibiotic resistance, by using the Firth’s penalized logistic regression, various helpful management adjustments could be identified, like regular monitoring of feed and water, especially when using water from one’s own well, following climatic recommendations or retrofitting of a cooling system or preventive measures to reduce the use of antibiotics. In conclusion, it seems possible to improve the health status and reduce the frequency of antibiotic treatments in broiler flocks by adjusting management measures, which should lead to a reduced occurrence of antibiotic-resistant, opportunistic pathogens in the broilers’ environment.

## Introduction

According to the annual *scientific report on antibiotic consumption and treatment frequency* published by the German Federal Institute for Risk Assessment (**BfR**), the antibiotic treatment frequency (**TF**) for fattening broilers increased from 2015 to 2019 on the farm basis and has stagnated for the last 3 years showing median values of over 20 days per half year (Flor and Tenhagen, 2025). The TF is a German indicator, which states the number of therapy days of an average number of animals per flock and is calculated by the sum of treated animals per half a year multiplied by the treated days, divided by the average number of kept animals per half a year (Flor and Tenhagen, 2025). It is calculated every half year, published annually by the BfR, and allows for a comparison of different farms. However, these findings of stagnating TF for fattening broilers are contrary to the national resistance strategy DART 2030 (Bundesministerium für Gesundheit (BMG), 2024), which aims to reduce antibiotic resistance in human and veterinary medicine in Germany. Especially hospital-acquired colonizations or infections with resistant pathogens (Agyeman et al., 2022; Ayobami et al., 2022; European Centre for Disease Prevention and Control (EDCD), 2025), or residues of antibiotics (Shen et al., 2023) or resistant, opportunistic pathogens (Lupo et al., 2014; Savin et al., 2021) in the environment, pose a major risk for human health, leading to national and international strategies, such as DART 2030. Especially in broilers, it is well known that resistant microorganisms occur in the animal’s environment (Heinemann et al., 2020; Laube et al., 2014), which can spread through various vectors (Graham et al., 2009) and therefore pose a major risk to human health (van den Bogaard et al., 2001; Kluytmans et al., 2013). Bailey et al. (1994) and Dierikx et al. (2013) showed that day-old chicks, arriving from the hatchery, introduce resistant microorganisms into the cleaned and disinfected broiler houses. These microorganisms can subsequently cause infections in hatchlings and young broilers (Mast and Goddeeris, 1999; Song et al., 2021), allowing them to persist in critical sites like the spray cooling systems (Heinemann et al., 2020), drinking cups or floor cracks (Luyckx et al., 2015) and, thus, pose a risk for following flocks. Besides that, there is also a risk that resistant microorganisms colonize humans who work with broilers carrying such opportunistic pathogens (van den Bogaard et al., 2001), or who prepare/consume contaminated meat (Kluytmans et al., 2013). Besides these direct transmission ways, a spread of antibiotic-resistant bacteria also occurs through process water, e.g., in slaughterhouses and other actors in the value chain (Savin et al., 2021). Therefore, it is necessary to reduce the use of antibiotics to curb the spread of resistant microorganisms.

One aspect of DART 2030 aimed at reducing the occurrence of resistant microorganisms is the improvement of animal health status by adjusting their husbandry and management systems (BMG, 2024), a strategy that has already been confirmed in several studies on various aspects of management adjustments. As already mentioned, an inclusion of resistant microorganisms already occurs in day-old chickens, which is likely due to vertical transmission via the parent animals (Dórea et al., 2010). Mezhoud et al. (2016) showed that egg-shells can already be contaminated with antibiotic-resistant bacteria. To reduce this risk of spreading in broiler parent farms, vaccination programs could be implemented, as Dórea et al. (2010) investigated Salmonella vaccination on pullets during their rearing and showed a significantly lower occurrence of Salmonella in environmental samples, such as dust and carcasses from vaccinated pullet offspring. Besides that, management adjustments on broiler farms, such as changes in the production system, could reduce environmental levels of antimicrobial resistance (Sapkota et al., 2014; Younessi et al., 2022). Although the mean counts of coliforms and *Escherichia coli* (***E. coli***) were higher in antimicrobial-free farms, the value of antibiotic-resistant coliforms and multidrug-resistant *E. coli* exceeded in industrial compared to antimicrobial-free farms (Younessi et al., 2022). Similar results were shown in Sapkota et al. (2014), who compared the occurrence of *Salmonella* in conventional and organic poultry farms, determining higher values of *Salmonella* in organic farms but lower resistance occurrence. In this context, the breeds used and the stocking density are also often relevant. Comparing slow- and fast-growing breeds, a higher occurrence of multiresistant *E. coli* was detectable in the second group at the beginning of the fattening (Montoro-Dasi et al., 2020). The colonisation of one-day-old chickens with antibiotic-resistant, opportunistic pathogens could confirm vertical transmission via the parent animals. During the fattening period, the occurrence of resistant strains equalized, with equal values between the groups at the end of fattening. However, it can be confirmed that slow growing breeds are more robust against environmental influences and generally more active (Baxter et al., 2021; Singh et al., 2021), which could lower the risk of health problems and infections. Also, stocking density has a major impact on broilers’ health status, as shown in Buijs et al. (2009) and Abudabos et al. (2013). Abudabos et al. (2013) showed that lower performance was associated with higher density, which could be explained by a lower feed intake due to poorer access to feed. However, in Buijs et al. (2009), the final body weight failed to be affected by the stocking density, and also, the German *Animal Welfare Farm Animal Ordinance* (**TierSchNutztV** §19(3), Bundesministerium der Justiz und für Verbraucherschutz (BMJV) and Bundesamt für Justiz (BfJ), 19.01.2021) regulates the risk of poor access to feed by limiting the feeder per kilogram of total live weight. Besides that, animal welfare could be improved by lowering the stocking density, which may reduce the occurrence of footpad and hock dermatitis (Buijs et al., 2009) or excessively high body temperatures and stress (Abudabos et al., 2013).

In addition to modifications of the overall rearing system, management adjustments can also improve the health status of the animals and the reduce occurrence of opportunistic pathogens in the environment. Therefore, the feeding hygiene plays a major role, as shown in Alali et al. (2010) and Sapkota et al. (2014), where the feed was contaminated with *Salmonella*, which resulted in significantly higher occurrence of *Salmonella* in feces samples of broilers (Alali et al., 2010). Nevertheless, high-quality feed, the type of feed (Bjerrum et al., 2005) or the use of specific additives (Salim et al., 2013; Michalczuk et al., 2021; Vlaicu et al., 2021; Zhang et al., 2021) can also improve the health status of broilers.

Overall, it is evident that the management system of a broiler farm can strongly influence the welfare and health status of the broilers by affecting the microbial load and the occurrence of resistant microorganisms (i.e., methicillin-resistant *S. aureus* (**MRSA**) and species with resistance to extended-spectrum beta-lactams (carbapenem, cephalosporine, penicillin)) in their environment. To provide practical recommendations for management adjustments, it is necessary to first consider the current practices implemented on broiler farms. However, to evaluate multiple different farms in a wide range, a concept is necessary to examine multiple farms from different locations. Therefore, the aim of this study was to implement an analytical approach to evaluate and compare various broiler farms, Germany-wide, with respect to management practices, preventive measures, and the occurrence of resistant microorganisms in the broiler environment, in order to identify relevant risk factors and potential management adjustments that could reduce the prevalence of antibiotic-resistant, opportunistic pathogens.

## Material and methods

### Conceptual framework, call for participation and ethics of the study

Data collection and sampling occurred in Germany between January 2024 and February 2025. The farmers were recruited through trade fair appearances, specialist lectures, individual letters and distribution of the online survey via QR code. Data collection complied with the guidelines of the Technical University of Munich. Furthermore, farmers were informed of the data protection guidelines and the voluntary nature of participation in the study in the cover letter and on the introduction page of the online survey, which were further confirmed by signing a data transfer declaration. To allocate farmers’ answers from the survey to the environmental samples collected in their broiler house, farmers were assigned a unique, but non-traceable ID, which they provided for both parts of the study (survey and samples).

It has to be mentioned that the aim of this study was to classify and analyse at least 20 farms with various management practices, visiting the farms by a research team. Due to the ongoing risk of avian influenza and the spread of other opportunistic pathogens in the broiler flock, we assume that farmers’ willingness to participate was very low. Even though the methodical approach was adjusted by changing from visiting the farm by the research team to an individual sampling of the broiler houses by the farmer and postal shipping of the samples, the goal to result in a higher number of responses to reach our target of 20 farms failed. To recruit participants, farms for trainees (*n* = 69) and participants from previous projects (*n* = 31) throughout Germany were contacted and inquired by telephone. Additionally, we contacted various interfaces, including veterinarians (*n* = 10), public institutions (*n* = 5), fattening and poultry breeder associations (*n* = 3), qualification associations (*n* = 3), and alumni associations of agricultural universities (*n* = 1). We also spoke at public events and kindly requested participation, such as poultry conferences and the fair EuroTier 2024 in Hannover, and utilized private contacts. Overall, we distributed approximately 60 sample packages to farmers for sample collection after they agreed to participate, whereas only 14 complete sample packages were returned.

Due to the farm-specific information and management data collected in the survey, and the sensitive medical data from resistance testing, traceability cannot be ruled out, despite everything being documented anonymously. Therefore, raw data are only available from the authors after a reasonable request and signing a data consent form. Additionally, no ethical approval for the use of animals was required, as an examination of living animals or even contact between researchers and animals was excluded.

### Survey

An online survey was conducted to collect management data of the participating farmers using the licensed online survey tool UNIPARK (Tivian XI GmbH, Cologne, Germany) (supporting information 1, translated version). Since the sampling area was Germany, the survey was conducted in German and then translated into English for the purposes of this publication. Overall, nine different subsections were queried (contact (*n* = 4), personal data (*n* = 3), general operating data (*n* = 6, 2 filter questions included), fattening data (*n* = 8), management technical data (*n* = 9, 1 filter question included), housing climate (*n* = 8, 2 filter questions included), feeding system (*n* = 5, 2 filter questions included), health information (*n* = 8, 4 filter questions included), cleaning system (*n* = 4)), with 55 different questions, including single choice questions (*n* = 24, including 6 semi-open questions with additions to add one’s own, not included, answer), multiple choice, semi-open questions (*n* = 10), open questions (*n* = 15) and combined closed question with open specification (questions, providing predefined answer categories, while allowing to specify a more detailed answer within the selected category using a free-text field, e.g. the category (micronutrition) and type (proteins, carbohydrates, lipids) of feed additives, *n* = 6). The first two subsections contained questions about the participant (e.g., gender or level of education), followed by questions about the operating type, the type of poultry, or the number and level of education of the employees (general operating data). The section of fattening data included questions of the fattening itself, e.g., the used breeding line, the number of hygienic units (i.e., broiler houses with separate access) and animals per unit or the duration of the fattening, which was further enhanced by questions about the use of environmental enrichments, the wearing of protective clothing or the type of broiler house access (management technical data). The last sections asked detailed questions on the topic of climate conditions (e.g., the average temperature or relative humidity), the feeding system (e.g., numbers of feeding phases, origin of the dietary composition), the health information (e.g., the type of prophylactic measures to reduce the use of antibiotics or the decision criteria for using antibiotics) and the cleaning system (e.g., duration of the service period / down time between two broiler flocks or the commonly used cleaning or disinfectant agents). Only the question about the type of fattened poultry was a mandatory question, because the main type of poultry considered in this study was broiler. Additionally, some of these questions were filter questions, as, e.g., the use of environmental enrichments. If the farmer stated that he used enrichments, a subcategory asked which enrichments these were. Otherwise, this question was skipped.

### Environmental samples

#### Sampling

To reduce the risk of pathogen transmission and potential infection of the sampled broilers, a sampling approach was implemented in which the farmers collected the samples independently. This approach had the additional advantage of extending the sampling area to the whole of Germany by sending empty, pre-labelled bags and tubes to the farmers. The farmers were instructed to take pooled samples (manure, 250-500 g; water, ∼ 40 mL; dust, 1-5 g) of one of his or her hygienic broiler units during the last week of an average fattening period, and to ship them to the laboratory for further analysis on the same day of sampling.

Upon arrival in the lab, the samples were immediately cooled and processed by determining the DM of the manure, according to VDLUFA (2023) 3.1, the water pH (GS 842, Schott, Germany), turbidity (TIR210, VWR, Belgium) and electric conductivity (G1410, Greisinger, Germany) and the microbial load in all three types of samples.

#### Microbiological Analysis

Since the time frame on the farm between sampling and shipping differed between the participants, the occurrence of *Staphylococci* and *Staphylococcus aureus* (***S. aureus***), MRSA and species with resistance to extended-spectrum beta-lactams were only characterized qualitatively. Before analyzing, the manure and dust samples were diluted in a sterile physiologic saline solution (0.85 % saline; BR0053G, Oxoid Ltd., Thermo Fisher Scientific, USA; supplemented with 1 g/L tryptone; BIT1332, Apollo Scientific, UK) to 1:10 or 1:9-1:30 g/g solutions, respectively. It should be noted that the dust samples were very inhomogeneous, wherefore the dilution factor varied between 1:9 and 1:30, depending on the density of the dust sample. For manure, the 1:10 g/g solution were homogenized in filter bags, using a stomacher (BA 7021, Seward Ltd, UK). The water samples were directly analysed without further dilution.

*Staphylococci* and *S. aureus* were quantified using Baird-Parker-Agar (100063UA, VWR Chemicals, Belgium). Therefore, 100 µL of the aliquots were applied onto the agar by spread-plate technique and incubated (INE 550, Memmert GmbH + Co. KG, Germany) at 37°C for 24 h, then stored in the refrigerator for 24 h and assessed for the occurrence of *Staphylococc*i.

Detection of antibiotic-resistant bacteria was performed with CHROMagar pre-poured plates for MRSA after pre-enrichment (201402, MAST Diagnostica, Reinfeld, Germany) and ESBL (201407, MAST Diagnostica, Reinfeld, Germany) according to Savin et al. (2020), with dilution factors depending on the sample type and incubation at 37°C for 24 h. For MRSA pre-enrichment, 1 mL of the sample was diluted in 10 mL Mueller-Hinton broth (CM0405B, Oxoid Ltd., Thermo Fisher Scientific, USA; supplemented with 6.5 % NaCl) and incubated at 37°C for 24 h. In the next step 1 mL of the incubated Mueller-Hinton broth was pipetted in 10 mL tryptic soy broth (CM0129B, Oxoid Ltd., Thermo Fisher Scientific, USA), supplemented with aztreonam (50 mg/L; PHR1785-500MG, Merck KGaA, Germany) and cefoxitin (3.5 mg/L; BIM0108, Apollo Scientific, UK) and subsequent incubation of 24 h at 37°C. An aliquot of 100 µL was spread onto the plate and incubated for 24 h at 37°C. For further characterization, the individual colonies were selected from the selective plates based on their distinct morphology, incubated on Columbia agar with 5 % sheep blood (201190, MAST Diagnostica, Reinfeld, Germany) at 37°C for 18 to 24 h, and further identified using MALDI-TOF MS (Bruker Daltonics, Germany). The antibiotic susceptibility tests were conducted according to CLSI guidelines (M07-A10) by broth microdilution using Micronaut-S MDR MRGN-Screening system (MERLIN, Gesellschaft für mikrobiologische Diagnostika GmbH, Germany) (Savin et al. 2020). Tested antibiotics were aminoglycosides (amikacin), carbapenems (imipenem, meropenem), cephalosporins (cefotaxime, ceftazidime/avibactam, ceftologan/tazobactam), fluoroquinolones (ciprofloxacin, levofloxacin), penicillin (piperacillin, piperacillin/tazobactam, temocillin), tetracyclines (tigecycline), and miscellaneous agents (colistin, chloramphenicol, fosfomycin, trimethoprom/sulfamethoxazole). For the evaluation of the results, the clinical cut-off values from the European Committee on Antimicrobial Susceptibility Testing (EUCAST v. 15) (2025) were applied.

### Statistical analysis

Data were structured with Microsoft Excel (Version 2108) and statistically analysed with RStudio (4.5.0) (R core Team, 2025). All survey questions and microbiological analysis results were coded as binary values (0 and 1) for statistical analysis. Biofilm-producing detected species were defined by the presence of at least one of the bacteria known to be biofilm-forming in the water samples (*S. aureus*, resistant *Pseudomonas aeruginosa* or *Stenotrophomonas maltophilia*).

For the survey’s parameters, the answers were either in a binary order (e.g., access to an outdoor range or the fattening of a fast- vs. slow-growing broiler line) or the answers were split up (e.g., the type of bedding materials). For numerical answers, a limit was defined due to the magnitude (low vs. high average number of animals, with below or above 20,000 animals per flock, respectively) or based on recommendations (climate data according to the German Agricultural Society (Deutsche Landwirtschafts-Gesellschaft, DLG-Ausschuss Geflügel (2024)). This approach resulted in more parameters to be statistically analysed for the risk evaluation. However, the microbiological analysis was conducted qualitatively, so the results have already been set in a binary order (microorganism was present (1) or not (0)). Due to the small number of participating farmers, a risk evaluation was conducted using the Firth’s penalized logistic regression (RStudio-package: logistf; Heinze and Schemper (2002) and Puhr et al. (2017)). The associated odds ratios (**OR**) were calculated by exponentiation of the determined effect (β): OR = e^β^. According to that, Spearman correlations were determined in order to be able to evaluate statements regarding the risk assessment of individual predictors. Interactions between the predictors were classified as only slightly relevant based on the categorical classification in the survey. Overall, significant correlations between the operating system (conventional vs. organic) and various management measures could be detected, which led to a separate analysis with only the conventional operating farms being included (*n* = 10), using the same evaluation methods (Firth’s penalized logistic regression and Spearman correlations). For statistical analysis, the contact and personal data which were asked in the survey were excluded. Besides that, a Permutation test in which 10,000 permutations were performed was carried out comparing the numerical data from the conventional and organic operating farms, based on the significant results of the Spearman correlation analysis. A p-value below 0.1 was considered a trend, and below 0.05 as significant.

## Results

### Management measures in German broiler farms

**Fig. 1:**
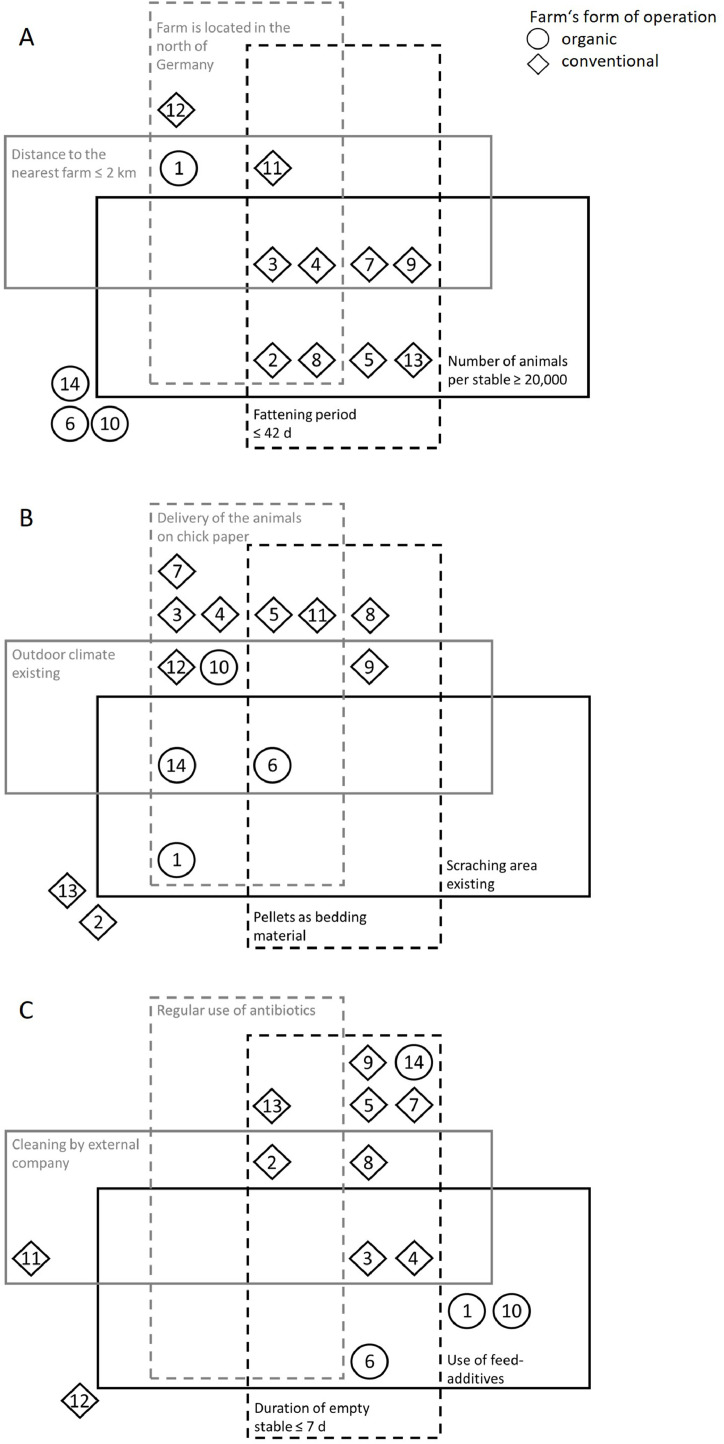
Various management measures of the surveyed farmers (*n* = 14), with numbers in the square means that the statement applies to the farm, otherwise it does not. A: Operational and fattening data, B: Management data, C: Cleaning and health data.

The surveyed farms showed heterogeneous management measures (Fig. 1), whereas a slight pattern emerged. Therefore, the organic operating farms (*n* = 4) had a slow growing line (mostly Hubbard JA 757, *n* = 3), with numbers of animals between 500 and 14,400 per fattening cycle and broiler house, whereas in conventional operating farms (*n* = 10), nine farmers fattened Ross 308 broilers with animal numbers ranging between 6,400 and 44,000 per broiler house. Based on this, the number of animals was significantly higher (*p* < 0.007) and the duration of the fattening period significantly lower (*p* < 0.001) in conventional operating farms (Tab. 1). Overall, 7 out of 14 farms were located in North Rhine-Westphalia or Lower Saxony (*n* = 4 and 3, respectively; defined as north of Germany in Fig. 1), the remaining 7 were from Bavaria (*n* = 6) or Baden-Württemberg (*n* = 1), with a distance to the next farm between 0.5 and 15 km. The most commonly used bedding material was straw pellets (*n* = 4), followed by short straw (*n* = 3). Less commonly used bedding materials, such as spelt husks or peat, were also employed (*n* = 1 each). Additionally, all farmers offered enrichment items, such as picking stones (*n* = 9), straw barns (*n* = 6), access to an outdoor range (*n* = 5) or a scratching area (*n* = 3). Broiler house temperature differed significantly between conventional and organic operating farms (*p* < 0.04). The feeding system was subdivided into at least three periods, where the dietary composition is determined by feed companies (*n* = 7), independently compiled (*n* = 4) or according to the specifications of the fattening association (*n* = 3). Additionally, the water was drawn from the public supply (*n* = 9) or from a farm-owned well (*n* = 5). The use of antibiotics varied between the individual farms, with three farmers using no antibiotics at all, eight in exceptional cases, one situation-dependent after consulting a veterinarian and two regularly at the broiler’s age of 1 to 4 days. TF were only given by seven farmers, with values from 0 to above 38 average treatment days during the last half year. The most commonly used antibiotics were aminoglycosides (*n* = 5) and lincosamides (*n* = 3), with 6 farmers restrained from using antibiotics (*n* = 3), as described, or skipped to provide the requested information (*n* = 3). Overall, every farmer uses vaccines as a method to reduce the use of antibiotics. In addition, the feed and water are monitored (*n* = 11 and 9, respectively), and feed or bedding additives were used (*n* = 6 and 1, respectively). After the fattening period, the broiler houses were cleaned and disinfected by the farmer (*n* = 9) or by a company (*n* = 5), with a duration of the service period of 5 to 28 days. Six of the farms use a combination of alkaline and acid cleaning products, while three use only an alkaline product for cleaning, or hydrogen peroxide, an acid or no cleaning product at all (*n* = 1 each). Disinfection of the broiler house was carried out with peroxide-containing (*n* = 8), acidic (*n* = 7), aldehyde- (*n* = 2) or iodine-containing (*n* = 2) products.

**Table 1:** Comparison of numerical data between conventional (*n* = 10) and organic (*n* = 4) operating farms carried out using Permutation test.

	Conventional		Organic		p value
	Mean	SE	Mean	SE	
Analytical parameters					
Water pH	7.1	0.1	7.5	0.2	0.036
Water electrical conductivity, µS	350	47	544	175	0.159
Water turbidity, NTU	2.2	1.0	1.1	0.5	0.594
DM manure, %	63.1	2.9	63.7	5.4	0.918
General operating data					
Distance next poultry farm, km	3.9	1.4	3.0	0.8	0.794
Fattening data					
Hygienic unit on the farm, n	1.8	0.4	1.5	0.3	0.856
Number of chicks per flock, n	25,960.0	3,557.3	5,375.0	3,140.2	0.005
Number of flocks per year, n	7.1	0.2	8.3	1.9	0.400
Duration of fattening period, days	41.8	1.5	78.3	12.6	0.001
Housing climate					
Average temperature,°C	26.4	0.6	21.3	2.4	0.030
Minimum temperature,°C	20.3	0.5	10.3	4.7	0.003
Maximum temperature,°C	34.5	0.3	36.3	1.7	0.163
Average relative humidity, %	61.8	2.2	61.5	6.5	0.947
Minimum relative humidity, %	50.9	2.1	52.5	7.5	0.735
Maximum relative humidity, %	73.9	3.2	70.0	5.0	0.556
Cleaning system					
Duration of service period, days	7.1	0.4	13.5	5.0	0.049

### Occurrence of antibiotic-resistant microorganisms in environmental samples

*Staphylococci* and *S. aureus* were found in all dust and manure samples, while water samples showed either none (*n* = 3), only *Staphylococci* (*n* = 5) or *S. aureus* (*n* = 1) or both (*n* = 5). Additionally, MRSA was absent from all environmental samples. In samples of 5 farms, no target antibiotic-resistant, opportunistic pathogens were detectable. In each of the remaining 9 farms, at least one phenotypically different target species was isolated from a selective CHROMagarESBL plates and was subsequently subjected to the antimicrobial susceptibility testing (Table 2). Since a different number of phenotypically different microorganism colonies occurred on the CHROMagarESBL plates, the listed species represent the minimum of variety of antibiotic-resistant, opportunistic pathogens of the individual farms instead of the number of collected samples on the farm. Accordingly, six farms yielded a total of ten antibiotic-resistant isolates classified as *Enterobacteriaceae* (Table 2), of which six were identified with resistance to extended-spectrum beta-lactams and the remaining four as multidrug-resistant Gram-negative bacteria resistant to three antibiotic classes (**3MDRO**). One of 2 isolates of *Acinetobacter baumannii-calcoaceticus* complex (**ABC complex**) showed resistance to 4 of 5 antibiotic classes resistance according to EUCAST (2025), which could be classified as multidrug-resistant (**MDR**) according to Magiorakos et al. (2012).

**Table 2:** Results of the antimicrobial susceptibility test according to EUCAST (2025), carried out in the different species isolated in environmental samples (water, dust, manure) of broiler farms (*n* = 14), during the last week of an average fattening cycle. Sorted by farm number.

White, antibiotic not recommended for use in the respective species according to EUCAST (2025); grey, differentiation of the species only in wildtype* (-) or non-wildtype (+) according to EUCAST (2025); green, sensitive; orange, intermediate; red, resistant; bold, relevant antibiotic to identify species with resistance to extended-spectrum beta-lactams and multiresistant gram-negative bacteria to 3 (3MDRO) or 4 antibiotic agents (4MDRO). D, dust; M, manure; W, water.

*wildtype/non-wildtype: species, which could only be defined as "susceptible, increased exposure” (EUCAST, 2025) and could only be differentiated into intermediate or resistant (due to an occurred mutation).

Due to the methodology for sample collection used, where the farmers collected the samples individually in the last week of a fattening system and shipped the samples to the laboratory by post, the duration between sample collection and laboratory processing (in days) was added in the statistical analysis. Therefore, this duration of processing showed a negative correlation with the occurrence of extended-spectrum beta-lactams in dust samples (*p* < 0.03, *r* = −0.605), *Enterobacteriaceae* (*p* < 0.05, *r* = −0.539), *E. coli* (*p* < 0.02, *r* = −0.650) and MDR (*p* < 0.05, *r* = −0.539).

### Risk evaluation of different management measures

**Fig. 2:**
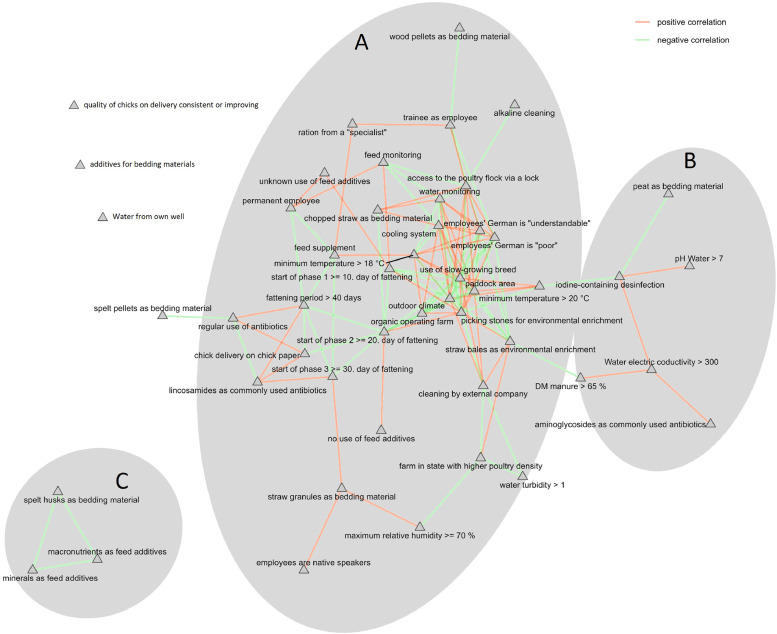
Kamada-Kawai network of the identified relevant predictors of all farms (conventional and organic operating farms, *n* = 14) according to the determined Firth’s penalized logistic regression and the Spearman correlations. Shown are significant correlations (*p* < 0.05) associated with operating system (A), drinking water hygiene (B), or alternative strategies (C).

Based on the 7 categories, which were asked in the survey (contact and personal data excluded), every category showed significant effects on various environmental and the risk of colonization of the broilers (Table 3, Table 4, Table 5). The predictor with the most common significant effect (*p* < 0.05) on various tested parameters was the start of the second feeding phase (*n* = 4 positive effects), followed by the fattening of a slow-growing breeding line (*n* = 3 positive effects), the access to an outdoor range (*n* = 3 positive effects), the existence of outdoor climate in the broiler house (*n* = 3 positive effects), the availability of picking stones (*n* = 3 negative effects), the start of the third feeding phase (*n* = 3 positive effects), or aminoglycoside as commonly used antibiotic (*n* = 3 negative effects). Based on the identified risk-related effects, the Spearman correlations showed high correlations, according to the topics “operating system”, “drinking water hygiene” and “alternatives for antibiotic use” (Fig. 2).

**Table 3:** Risk effects of various management strategies according to the Firth’s penalized logistic regression on the occurrence of bacterial species in water samples All farms were included (*n* = 14), and only the tendential (0.05 < *p* < 0.1) and significant (*p* < 0.05) effects were shown.

Predictors	Effects	Lower CI	Higher CI	Odd ratios	p-values
*Staphylococcus aureus* in water samples					
Permanent employees	2.147	−0.303	5.168	8.556	0.088
Straw granules as bedding material	2.565	−0.014	7.546	13.000	0.051
Spelt husks as bedding material	−2.833	−7.837	−0.174	0.059	**0.035**
Ration from a "specialist"	−2.565	−7.546	0.014	0.077	0.051
Alkaline cleaning	2.197	0.025	4.886	9.000	**0.047**
*Staphylococcus spp.* in water samples					
Employees' German is "understandable" 1	2.833	−0.489	8.032	17.000	0.095
Employees' German is "poor" 1	2.833	−0.489	8.032	17.000	0.095
Access to the poultry flock via a lock	−3.045	−8.101	−0.199	0.048	**0.035**
Straw bales as environmental enrichment	3.421	0.767	8.443	30.600	**0.009**
Biofilm-producing detected species in water samples ^2^					
Organic operating farm	2.693	0.320	5.676	14.778	**0.025**
Use of low-growing breed 1	2.071	−0.169	4.783	7.933	0.071
Picking stones for environmental enrichment 1	−2.071	−4.783	0.169	0.126	0.071
Outdoor range available 1	2.071	−0.169	4.783	7.933	0.071
Outdoor climate available 1	2.071	−0.169	4.783	7.933	0.071
Minimum temperature > 18°C 1	−3.892	−9.003	−1.048	0.020	**0.005**
Cooling system available 1	−3.045	−8.101	−0.199	0.048	**0.035**
Ration from a "specialist"	−2.693	−5.676	−0.320	0.068	**0.025**
Start of phase 2 ≤ 20. day of fattening 1	2.565	−0.014	7.546	13.000	0.051
Feed supplement to minimize antibiotic use	3.421	0.767	8.443	30.600	**0.009**
Feed monitoring to minimize antibiotic use	−2.071	−4.783	0.169	0.126	0.071

Bold, significant Firth’s penalized logistic regressions. CI, Confidence interval.

1 significant (*p* < 0.05) correlations with the operating system. ^2^ defined by the presence of at least one of the bacteria known to be biofilm-forming in the water samples (*S. aureus*, resistant *Pseudomonas aeruginosa*, or *Stenotrophomonas maltophilia*).

**Table 4:** Risk effects of various management strategies according to the Firth’s penalized logistic regression on the appearance of species with resistance to extended-spectrum beta-lactams in water, dust and manure samples. All farms were included (*n* = 14), and only the tendential (0.05 < *p* < 0.1) and significant (*p* < 0.05) effects were shown.

Predictors	Effects	Lower CI	Higher CI	Odd ratios	p-values
Water samples					
Water pH > 7	2.071	−0.169	4.783	7.933	0.071
Use of low-growing breed 1	2.599	0.036	7.577	13.444	**0.046**
Straw granules as bedding material	−2.071	−4.783	0.169	0.126	0.071
Picking stones for environmental enrichment 1	−2.599	−7.577	−0.036	0.074	**0.046**
Outdoor range available 1	2.599	0.036	7.577	13.444	**0.046**
Outdoor climate available 1	2.599	0.036	7.577	13.444	**0.046**
Maximum relative humidity ≥ 70	2.398	0.024	5.390	11.000	**0.048**
Unknown use of feed additives	−4.007	−9.865	−0.533	0.018	**0.021**
Start of phase 1 ≤ 10. day of fattening	2.833	0.527	5.793	17.000	**0.015**
Start of phase 2 ≤ 20. day of fattening 1	2.197	0.025	4.886	9.000	**0.047**
Dust samples					
Fattening period over 40 days	2.959	0.360	7.953	19.286	**0.023**
Water from own well	2.960	0.360	7.952	19.286	**0.023**
Chopped straw as bedding material	2.959	0.360	7.953	19.286	**0.023**
Minimum temperature > 18°C 1	−2.457	−7.447	0.176	0.086	0.070
Start of phase 2 ≤ 20. day of fattening 1	2.255	0.110	4.933	9.533	**0.039**
Start of phase 3 ≤ 30. day of fattening	3.353	0.707	8.374	28.600	**0.010**
Feed supplement to minimize antibiotic use	2.255	0.110	4.933	9.533	**0.039**
Water monitoring to minimize antibiotic use	−2.457	−7.447	0.176	0.086	0.070
Lincosamides as commonly used antibiotics	−2.398	−7.417	0.322	0.091	0.088
Manure samples					
Water electrical conductivity > 300	2.608	−0.182	7.635	13.571	0.068
Peat as bedding material	−3.219	−8.408	0.080	0.040	0.056
Spelt husks as bedding material	−3.219	−8.408	0.080	0.040	0.056

Bold, significant Firth’s penalized logistic regressions. CI, Confidence interval.

1 significant (*p* < 0.05) correlations with the operating system.

**Table 5:** Risk effects of various management strategies according to the Firth’s penalized logistic regression on the occurrence of resistant microorganisms and resistance patterns in all environmental samples (water, dust or manure). All farms were included (*n* = 14), and only the tendential (0.05 < *p* < 0.1) and significant (*p* < 0.05) effects were shown.

Predictors	Effects	Lower CI	Higher CI	Odd ratios	p-values
*Enterobacteriaceae*^1^					
Water pH > 7	2.071	−0.169	4.783	7.933	0.071
Water turbidity > 1	−2.197	−4.886	−0.025	0.111	**0.047**
Use of low-growing breed ^2^	2.599	0.036	7.577	13.444	**0.046**
Fattening period over 40 days	2.071	−0.169	4.783	7.933	0.071
Picking stones for environmental enrichment ^2^	−2.599	−7.577	−0.036	0.074	**0.046**
Outdoor range available ^2^	2.599	0.036	7.577	13.444	**0.046**
Outdoor climate available ^2^	2.599	0.036	7.577	13.444	**0.046**
Start of phase 2 ≤ 20. day of fattening ^2^	2.197	0.025	4.886	9.000	**0.047**
Start of phase 3 ≤ 30. day of fattening	2.708	0.386	5.676	15.000	**0.021**
Regular use of antibiotics (on fattening days 1-4)	−2.608	−7.635	0.182	0.074	0.068
Aminoglycosides as commonly used antibiotics	−2.398	−5.390	−0.024	0.091	**0.048**
Iodine-containing disinfectant	−2.608	−7.635	0.182	0.074	0.068
*Enterobacter hormaechei*^1^					
DM manure > 65 %	2.608	−0.182	7.635	13.571	0.068
Employees are native speakers	2.833	−0.489	8.032	17.000	0.095
No use of feed additives	−2.608	−7.635	0.182	0.074	0.068
Start of phase 3 ≤ 30. day of fattening	2.497	−0.304	7.526	12.143	0.083
Cleaning by external company	−2.608	−7.635	0.182	0.074	0.068
*Escherichia coli*^1^					
Water pH > 7	2.693	0.320	5.676	14.778	**0.025**
Water electrical conductivity > 300	2.071	−0.169	4.783	7.933	0.071
Start of phase 3 ≤ 30. day of fattening	1.946	−0.310	4.666	7.000	0.092
Aminoglycosides as commonly used antibiotics	−3.664	−8.774	−0.835	0.026	0.008
Iodine-containing disinfectant	−3.045	−8.101	−0.199	0.048	**0.035**
*Klebsiella pneumoniae*^1^					
Spelt pellets as bedding material	−4.394	−10.282	−0.704	0.012	**0.019**
Regular use of antibiotics (on fattening days 1-4)	−3.219	−8.408	0.080	0.040	0.056
*Actinetobacter baumannii/calcoaceticus complex*^1^					
Water electrical conductivity > 300	2.608	−0.182	7.635	13.571	0.068
Spelt pellets as bedding material	−3.219	−8.408	0.080	0.040	0.056
Use of additives for bedding materials	−3.219	−8.408	0.080	0.040	0.056
*Pseudomonas aeruginosa*^1^					
Employed trainees	−2.833	−8.032	0.489	0.059	0.095
Chick delivery on chick paper	3.045	0.199	8.101	21.000	**0.035**
Wood pellets as bedding material	−3.219	−8.408	0.080	0.040	0.056
No use of feed additives	−2.608	−7.635	0.182	0.074	0.068
Start of phase 1 ≤ 10. day of fattening	2.608	−0.182	7.635	13.571	0.068
*Stenotrophomonas maltophilia*^1^					
Water pH > 7	2.693	0.320	5.676	14.778	**0.025**
Straw granules as bedding material	−2.693	−5.676	−0.320	0.068	**0.025**
Maximum relative humidity ≥ 70	3.664	0.835	8.774	39.000	**0.008**
Unknown use of feed additives	−4.007	−9.865	−0.533	0.018	**0.021**
Start of phase 1 ≤ 10. day of fattening	2.071	−0.169	4.783	7.933	0.071
Start of phase 3 ≤ 30. day of fattening	1.946	−0.310	4.666	7.000	0.092
Feed supplement to minimize antibiotic use	2.565	−0.014	7.546	13.000	0.051
Multiresistant gram-negative bacteria (3MDRO)					
Quality of chicks on delivery consistent or improving	−3.555	−9.436	−0.001	0.029	**0.050**
Minimum temperature > 20°C	3.555	0.001	9.436	35.000	**0.050**
Multidrug-resistant bacteria					
Water pH > 7	2.071	−0.169	4.783	7.933	0.071
Water turbidity > 1	−2.197	−4.886	−0.025	0.111	**0.047**
Use of low-growing breed ^2^	2.599	0.036	7.577	13.444	**0.046**
Fattening period over 40 days	2.071	−0.169	4.783	7.933	0.071
Picking stones for environmental enrichment ^2^	−2.599	−7.577	−0.036	0.074	**0.046**
Outdoor range available ^2^	2.599	0.036	7.577	13.444	**0.046**
Outdoor climate available ^2^	2.599	0.036	7.577	13.444	**0.046**
Start of phase 2 ≤ 20. day of fattening ^2^	2.197	0.025	4.886	9.000	**0.047**
Start of phase 3 ≤ 30. day of fattening	2.708	0.386	5.676	15.000	**0.021**
Regular use of antibiotics (on fattening days 1-4)	−2.608	−7.635	0.182	0.074	0.068
Aminoglycosides as commonly used antibiotics	−2.398	−5.390	−0.024	0.091	**0.048**
Iodine-containing disinfectant	−2.608	−7.635	0.182	0.074	0.068

Bold, significant Firth’s penalized logistic regressions. CI, Confidence interval.

1 Isolated from a selective CHROMagarESBL plates and was subsequently subjected to antimicrobial susceptibility testing. ^2^ significant (*p* < 0.05) correlations with the operating system.

**Fig. 3:**
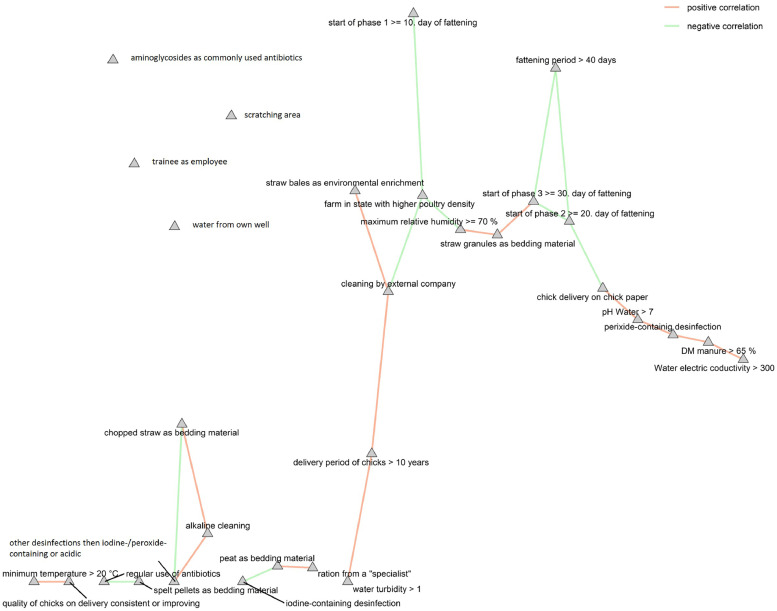
Kamada-Kawai network of the identified relevant predictors of only the conventional operating farms (*n* = 10) according to the determined Firth’s penalized logistic regression and the Spearman correlations. Shown are significant correlations (*p* < 0.05).

According to the bundled correlations associated with the operating system, the organic operating farms (*n* = 4) were excluded for a second, similar statistical analysis. In this statistical analysis, the parameters for the use of spelt pellets as bedding material (*n* = 2 negative effects), a maximum of above 70 % of relative humidity at the end of the fattening period (*n* = 2 positive effects), and no knowledge about adding feed additives (*n* = 2 negative effects) showed the most significant effects (Tab. 6). Contrary to Fig. 2, the Kamada-Kawai network of the Spearman correlations shows relevant parameters according to the statistical analysis of the farms, excluding the organic operating ones, showed noticeably fewer junctions (Fig. 3).

**Table 6:** Risk effects of various management strategies according to the Firth’s penalized logistic regression on the occurrence of bacterial species in water samples, species with resistance to extended-spectrum beta-lactams in water, dust and manure samples and the occurrence of microorganisms and resistance patterns in all environmental samples (water, dust or manure). Only conventional farms were included (*n* = 10), and only the tendential (0.05 < *p* < 0.1) and significant (*p* < 0.05) effects were shown.

Predictors	Effects	Lower CI	Higher CI	Odd ratios	p-values
*Staphylococcus aureus* in water samples					
Water turbidity > 1	2.197	−0.222	5.208	9.000	0.076
Straw granules as bedding material	2.734	−0.052	7.786	15.400	0.055
Alkaline cleaning	2.734	−0.052	7.786	15.400	0.055
*Staphylococcus spp*. in water samples					
Straw bales as environmental enrichment	4.654	1.326	10.476	105.000	**0.004**
Cleaning by external company	−2.734	−7.786	0.052	0.065	0.055
Biofilm-producing detected species in water samples 1					
Peat as bedding material	4.043	0.334	9.937	57.000	**0.032**
Ration from a "specialist"	−2.833	−8.032	0.489	0.059	0.095
Iodine-containing disinfection	2.833	−0.489	8.032	17.000	0.095
species with resistance to extended-spectrum beta-lactams in water samples					
Straw granules as bedding material	−2.734	−7.786	0.052	0.065	0.055
Maximum relative humidity ≥ 70 %	3.296	0.403	8.423	27.000	**0.023**
Unknown use of feed additives	−3.555	−9.436	−0.001	0.029	0.050
Start of phase 1 ≥ 10. day of fattening	2.197	−0.222	5.208	9.000	0.076
species with resistance to extended-spectrum beta-lactams in dust samples					
Fattening period > 40 days	2.785	0.027	7.831	16.200	**0.048**
Water from own well	3.412	0.518	8.528	30.333	**0.018**
Chopped straw as bedding material	2.565	−0.328	7.635	13.000	0.085
Start of phase 2 ≥ 20. day of fattening	2.147	−0.303	5.168	8.556	0.088
Start of phase 3 ≥ 30. day of fattening	3.497	0.642	8.614	33.000	**0.013**
Other disinfection (than iodine-/peroxide/acidic)	2.565	−0.328	7.635	13.000	0.085
species with resistance to extended-spectrum beta-lactams in manure samples					
Water electric conductivity> 300	2.565	−0.328	7.635	13.000	0.085
Peat as bedding material	−2.833	−8.032	0.489	0.059	0.095
Spelt pellets as bedding material	−2.833	−8.032	0.489	0.059	0.095
*Enterobacteriaceae*^1^					
Water turbidity > 1	−2.197	−5.208	0.222	0.111	0.076
Water from own well	2.734	−0.052	7.786	15.400	0.055
Start of phase 3 ≥ 30. day of fattening	2.197	−0.222	5.208	9.000	0.076
*Enterobacter hormaechei*^1^					
DM manure > 65 %	2.565	−0.328	7.635	13.000	0.085
Delivery period of chicks > 10 years	3.219	0.215	8.365	25.000	**0.035**
Air quality is tested	2.565	−0.328	7.635	13.000	0.085
No use of feed additives	−2.565	−7.635	0.328	0.077	0.085
*Escherichia coli*^1^					
Water pH > 7	2.147	−0.303	5.168	8.556	0.088
Water electric conductivity > 300	2.147	−0.303	5.168	8.556	0.088
Aminoglycosides as commonly used antibiotics	−3.296	−8.423	−0.403	0.037	**0.023**
Iodine-containing disinfection	−2.565	−7.635	0.328	0.077	0.085
*Klebsiella pneumoniae*^1^					
Spelt pellets as bedding material	−4.043	−9.937	−0.334	0.018	**0.032**
Regular use of antibiotics (on fattening days 1-4)	−2.833	−8.032	0.489	0.059	0.095
*Actinetobacter baumannii/calcoaceticus complex*					
Spelt pellets as bedding material	−4.043	−9.937	−0.334	0.018	**0.032**
Regular use of antibiotics (on fattening days 1-4)	−2.833	−8.032	0.489	0.059	0.095
*Pseudomonas aeruginosa*^1^					
Employed trainees	−3.807	−9.707	−0.081	0.022	**0.045**
Hygienic unit on the farm > 1	−2.565	−7.635	0.328	0.077	0.085
Chick delivery on chick paper	2.565	−0.328	7.635	13.000	0.085
Wood pellets as bedding material	−2.833	−8.032	0.489	0.059	0.095
Scratching area available	−2.833	−8.032	0.489	0.059	0.095
No use of feed additives	−2.565	−7.635	0.328	0.077	0.085
Peroxide-containing disinfection	2.565	−0.328	7.635	13.000	0.085
*Stenotrophomonas maltophilia*^1^					
Water pH > 7	2.147	−0.303	5.168	8.556	0.088
Farm in a state with a higher poultry density	2.147	−0.303	5.168	8.556	0.088
Straw granules as bedding material	−3.412	−8.538	−0.518	0.033	**0.018**
Maximum relative humidity ≥ 70 %	4.595	1.285	10.415	99.000	**0.004**
Unknown use of feed additives	−3.555	−9.436	−0.001	0.029	**0.050**
Multiresistant gram-negative bacteria (3MDRO)					
Quality of chicks on delivery consistent or improving	−3.555	−9.436	−0.001	0.029	**0.050**
Minimum temperature > 20°C	3.555	0.001	9.436	35.000	**0.050**
Multidrug-resistant bacteria					
Water turbidity > 1	−2.197	−5.208	0.222	0.111	0.076
Water from own well	2.734	−0.052	7.786	15.400	0.055
Start of phase 3 ≥ 30. day of fattening	2.197	−0.222	5.208	9.000	0.076

Bold, significant Firth’s penalized logistic regressions. CI, Confidence interval.

1 Isolated from a selective CHROMagarESBL plates and was subsequently subjected to antimicrobial susceptibility testing. ^2^ defined by the presence of at least one of the bacteria known to be biofilm-forming in the water samples (*S. aureus*, resistant *Pseudomonas aeruginosa*, or *Stenotrophomonas maltophilia*).

## Discussion

This study aimed to implement an approach to examine and evaluate a broad range of broiler farms independently and, furthermore, to identify critical risk factors and potential adjustments to reduce the potential microbial contamination and occurrence of antibiotic-resistant, opportunistic pathogens in the broiler’s environment. Therefore, data from 14 German broiler farms were collected to evaluate and compare the various management measures with the occurrence of opportunistic pathogens in environmental samples. The sampling strategy was deliberately designed to enable the collection of samples from a large number of farms across Germany. Accordingly, the shipping procedure for transporting samples to the laboratory was chosen to ensure a practical and scalable approach—both for the current study and as a foundation for potential future monitoring with an even broader scope. The methodological limitations associated with this approach were consciously accepted, as they allow for a significantly larger sample size and thereby increase the overall value of the findings. As demonstrated by the negative correlation between shipping time and the detection of certain microorganisms, this methodology does not lead to an overestimation but rather to a slight underestimation of the microbial load of broiler farms. A limitation of the results is known due to the small sample size. Although great effort was made to achieve the target of 20 farms, the recruitment failed to be successful. However, the results presented provide a solid basis for discussion of measures within the broader scientific context and demonstrate a practical approach for larger-scale monitoring.

The management measures could be identified as heterogeneous, as shown exemplarily in Fig. 1, whereby the operating system seemed to divide the farms into two groups, characterizing various fattening parameters like the growing line, the number of animals or the existence of an outdoor range or outdoor climate. These findings could be confirmed with the determined Spearman correlations (*p* < 0.005) associated with the operating system. Additionally, the organic operating farms seem to have a higher maximum and lower minimum temperature in the broiler house, which could be attributed to the presence of an outdoor range or outdoor climate. Therefore, the temperature is subject to ambient fluctuations and is difficult to keep stable. The DLG recommends an optimal temperature at the beginning of the fattening of 32-33°C with a reduction during the fattening of 1°C-steps to 20°C (DLG-Ausschuss Geflügel, 2024). Excluding those farms that stated having outdoor climate (*n* = 5), 7 showed higher maximum temperatures, 5 showed higher minimum temperatures than recommended, and 3 were below the minimum temperature stated by the DGL.

A high scepticism seemed to occur among the farmers answering questions about the use of antibiotics, wherefore only 7 farms stated their TF. As already described, the TF indicates the number of therapy days of an average number of animals per flock (Flor and Tenhagen, 2025). Three of the farms showed a TF of 0, from which 2 of them were organic operating farms. Therefore, of the stated 4 organic operating farms, two did not use antibiotics at all (TF of 0), and two only used them in exceptional cases (these farmers did not provide any TF). However, the antibiotic treatment of broiler chickens in organic operating farms is not prohibited, and from an animal welfare perspective, it is even reprehensible not to treat animals appropriately (Animal Welfare Act, Bundesministerium der Justiz und für Verbraucherschutz (BMJV) and Bundesamt für Justiz (BfJ), 2023). However, it can be assumed that the use of antibiotics in organic operating farms is lower than in conventional ones. Additionally, the German Federal Office of Consumer Protection and Food Safety (Bundesamts für Verbraucherschutz und Lebensmittelsicherheit, **BVL**) published the national key values, which are upper limits for farms fattening more than 10,000 broilers on average, as an antibiotic reduction scheme (BVL, 2025). Based on that, 3 of the remaining farms were below the first limit of 23.093, whereas the farm with a TF of 38 was above the second limit of 32.974 and should take action (writing and submitting an action plan to reduce antibiotic use) to reduce the number of days antibiotics are used (BVL, 2025). It should also be noted that the farm with the highest TF was also one of the 2 farms that reported using antibiotics regularly within the first 4 days of life. The most commonly used antibiotic agents were aminoglycosides, followed by lincosamides, which matches the values of the most used classes of antibiotics published by the BfR (Flor and Tenhagen, 2025).

The isolated antibiotic-resistant opportunistic pathogens from environmental samples are consistent with other comparable studies. For example, Heinemann et al. (2020) also isolated *Enterobacteriaceae* like *Enterobacter spp*. or *Klebsiella pneumoniae* (***K. pneumoniae***) and *Acinetobacter baumannii* (***A. baumannii***) or *Pseudomonas aeruginosa* (***P. aeruginosa***) in a German broiler farm, showing a high variance of different antibiotic-resistance patterns, for example, various isolates of *Enterobacter spp*., *K. pneumoniae, P. aeruginosa*, as 3MDRO. *Enterobacteriaceae*, especially *E. coli, Salmonella* and *Klebsiella,* were also isolated in liver samples of broilers, which were fattened commercially or as “backyard chickens”, in Kamboh et al. (2018). This study was conducted in Pakistan, where the regulations on the use of antibiotics differed compared to the European standard, e.g., antibiotics were fed as a growth promoter in the commercial fattened broiler. However, compared to those results, the occurrence of resistance to ciprofloxacin was lower in the isolated *E. coli* and *K. pneumoniae* in this study (*n* = 3/6 *E. coli, n* = 1/2 *K. pneumoniae*, compared to 82.52 % in Kamboh et al. (2018)), and to cefotaxime and ceftazidime was higher in the isolated *Enterobacteriaceae* in this study (*n* = 6/6 *E. coli, n* = 2/2 *K. pneumoniae*). Additionally, van den Bogaard et al. (2001) and Younessi et al. (2022) isolated coliforms and *E. coli* in various poultry farms with different management systems, whereby in van den Bogaard et al. (2001) 10 of 45 isolates showed resistance to 5 or more antibiotic agents. Moreover, these antibiotic-resistant patterns were similar to those tested on isolated *E. coli* in broiler farmers and slaughterers, concluding a transmission from animals to humans. The occurrence of *Acinetobacter. spp.* and *P. aeruginosa* is also consistent with other studies, like Tigabie et al. (2025). Although the isolated *A. baumannii* and *P. aeruginosa* in Tigabie et al. (2025) showed resistance to antibiotics, which were also considered in this study, it should be noted that in Tigabie et al. (2025), the isolated bacteria were primarily found in laying hens flocks and one-day-old chickens, and less in broiler flocks. According to Siverajan et al. (2024), a primary entry point for *A. baumannii* in humans is the respiratory tract, where it can cause infections such as pneumonia and be fatal. Thus, from an occupational health and safety perspective, reducing this pathogen or at least its antibiotic resistance effect is essential. Additionally, *A. baumannii* showed a high recovery rate in raw poultry meat, which could cause critical infections in humans, according to Lupo et al. (2014).

By combining operational data with pathogen occurrence data and their antibiotic-resistant patterns, it was possible to identify both improving and deteriorating management measures. According to the general operating data, the operating system plays a major role in the occurrence and antibiotic resistance of various pathogens. Although in this study the operating system showed only a significant effect on the occurrence of biofilm-producing detected species in water samples, various other predictors, which correlated with the operating system, showed significant effects, like the existence of an outdoor range or outdoor climate. Comparable studies such as those by Sapkota et al. (2014) and Younessi et al. (2022), have shown similar results, where the number of antibiotic resistances in isolated pathogens was lower in antibiotic-free farms compared to commercial farms, but the prevalence of environmental microbial contamination was higher in the antibiotic-free farms. Although in this study, the number of identified antibiotic-resistant opportunistic pathogens was lower in organic operating farms, this operating schema could pose a higher risk of biofilm-producing detected species in drinking water. The microbial load of the environmental samples was impossible to determine in this study due to the methodological approach.

Another aspect of the general operating data was the location of the farm. A higher density of broiler farms is present in the north of Germany (Thobe et al., 2024), which resulted in a trend of a higher risk for *Stenotrophomonas maltophilia* (***S. maltophilia***) in this study by analysis without the organic operating farms. It could be hypothesised that an environmental transmission occurs via wind, dust or other vectors between various broiler houses and farms due to a higher density of pathogens in the environment, although the distance (in km) to the next farm affected none of the parameters. Using water from one's own well also causes a higher risk of the occurrence of various parameters, like species with resistance to extended-spectrum beta-lactams in dust samples, considering all 14 farms. These findings are confirmed by the results from Tigabie et al. (2025) and may be caused by a higher risk of undetected contamination of private wells due to fewer qualitative analyses than at public sources. Additionally, Maran et al. (2016) found differences in the microbial load of wells due to structural measures such as the type of construction or depth. Flooding or heavy rain also poses a higher risk of contamination (Pieper et al., 2021), especially near farms or manure deposits (Abraham and Wenderoth, 2005).

In the fattening data, the duration of one fattening period seemed to be one of the more influential factors. This predictor also correlated with the operating system and, therefore, with the existence of an outdoor range and an outdoor climate. When considering only the conventional operating farms, a period above 40 days still resulted in a higher occurrence of species with resistance to extended-spectrum beta-lactams in dust samples. An explanatory approach could be that the manure persists during the whole fattening period, which could cause a summation of opportunistic pathogens, and, therefore, a higher microbial load in the environment. Another possible explanation could be antibiotic residues in manure in early stages of the fattening period, due to a regular treatment of antibiotics during the first 4 days of the broilers’ life, which may cause a selective pressure. Shen et al. (2023) showed high residues of antibiotic agents up to 25 days in the litter of laying hens, which could confirm the hypothesis of selective pressure due to antibiotic residues in the litter and, thus, the occurrence of antibiotic-resistant opportunistic pathogens. The results of Baxter et al. (2021) and Singh et al. (2021) showed that slow growing breeds were more robust against environmental influences, which could offset the negative effect of a higher microbial load in the animal’s environment. However, more detailed empirical data for organic operating farms, especially regarding the extended fattening period, should be collected, and recommendations for farmers should be developed. This might include not only considering the handling of manure during such long periods of occupation, but also the climate conditions in the broiler house or nutritional recommendations, such as the duration and number of feeding phases, depending on the chosen genetic line of slow-growing broilers. A partial removal of the manure at critical places during the fattening period might reduce the risk of pathogenic summation. Additionally, trends and significantly positive effects of spelt and wood pellets could be identified in the statistical evaluation, both with and without the organic operating farms. Some positive effects of these materials include the high water-absorbency of pellets compared to non-processed materials (Liegsalz et al., 2025) or essential oils, such as those found in wood (Johnston et al., 2001).

A predictor of the climate systems was the presence of a cooling system, although this parameter correlated with the operating system, as only 2 organic operating farms did without a cooling system. The primary cooling system employed was a spray fogging system, which could lead to an increase in relative humidity in the broiler house and, consequently, pose a higher risk of opportunistic pathogens in the animal’s environment, particularly a higher occurrence of species with resistance to extended-spectrum beta-lactams, as shown in Heinemann et al. (2020). According to that, if the minimum temperature is above 20°C, the risk of the occurrence of 3MDRO is increased. Therefore, a cooling system in the broiler house is an advantage for the stable climate, whereby the recommendations of the DLG regarding climate conditions in the broiler house should be followed (DLG-Ausschuss Geflügel, 2024).

Finally, the type or usage of antibiotic agents, and the type of reducing management measures influenced the risk of the occurrence of various opportunistic pathogens or antibiotic-resistance patterns. Using antibiotics on a regular basis, a trend of an increased risk of *Enterobacteriaceae* or *K. pneumoniae* and *A. spp*. was observed in the analysis of this study's samples, regardless of whether organic operating farms were included or excluded. These relations could be seen as confirmation that the ban on the prophylactic use of antibiotics or their use as growth promoters is considered important to reduce the risk of the occurrence of antibiotic resistant bacteria (European Parliament, Council of the European Union (25-26), 2003). Additionally, considering all participating farms, monitoring the feed or water showed trends to reduce the risks of opportunistic pathogens in the environment, while the reported supplementation of feed additives was associated with increased risk.

As mentioned, the samples were shipped to the laboratory by post, which indicates a relatively long period during which the cooling could not be ensured. This might have resulted in a shift of the microbial pattern within the samples. Also, fungi, which were not the focus of this study, may have suppressed some present bacteria, or some did not survive over time. Therefore, it can be assumed that the quantities or occurrences of various antibiotic-resistant microorganisms have been underestimated, but not overestimated. Furthermore, the low number of participating farms may have resulted in different degrees of significant effects compared to a larger sample size. Thus, it is possible, that, with a larger number of participants, less significant effects may appear, but, the conducted methodology appears to be a promising way to evaluate farms on a large-scale basis, by instructing farmers to collect samples from the broilers’ environment themselves, without posing a risk of pathogen spread between the farms due to an external sampling team. Uniform sampling, using precise instructions and standardized sampling tools, is essential, but it seemed to be feasible with samples from the broiler house’s environment.

Overall, this study presented a first attempt to identify various predictors for the reduced or increased risk of antibiotic-resistant, opportunistic pathogens in the broilers’ environment by comparing existing management measures and the occurrence of various opportunistic pathogens or antibiotic-resistance patterns. This approach appears promising for larger sample sizes. In this example, different management measures could be observed between organic and commercial operation farms, as evidenced by fattening data, such as the average number of broilers per flock, the duration of the fattening period, or the animals’ access to an outdoor range. However, various management measures can be identified as influential in the occurrence of antibiotic-resistant, opportunistic pathogens in environmental samples from broiler farms. For instance, the climate conditions should be followed as recommended, or a cooling system could improve the conditions if operated according to good practice. Additionally, a regular monitoring of feed and water, especially when using water from one’s own well, or preventive measures, like changing the bedding materials, compliance with hygienic conditions, such as access via lock, or consulting a “specialist”, could also reduce the environmental microbial load to achieve the overall goal to improve the health status of broiler and reduce the number of antibiotic treatments.

## Funding sources

This work (Model- and Demonstration Project for animal welfare) is financially supported by the Federal Ministry of Agriculture, Food and Regional Identity (BMLEH) based on a decision of the Parliament of the Federal Republic of Germany, granted by the Federal Office for Agriculture and Food (BLE); grant number 2820MDT220.

## CRediT authorship contribution statement

**Theresa M Liegsalz:** Writing – original draft, Visualization, Validation, Software, Project administration, Methodology, Investigation, Formal analysis, Data curation, Conceptualization. **Mykhailo Savin:** Writing – review & editing, Methodology, Formal analysis, Data curation. **Céline Heinemann:** Writing – review & editing, Supervision, Methodology, Funding acquisition, Conceptualization. **Julia Steinhoff-Wagner:** Writing – review & editing, Visualization, Supervision, Resources, Project administration, Methodology, Funding acquisition, Formal analysis, Data curation, Conceptualization.

## Disclosures

We have no conflicts of interest to disclose, and the results have not been published in any other way, nor is the manuscript currently under consideration for publication. The current manuscript should be used for a doctoral project at the TUM School of Life Sciences. Additionally, the manuscript was written through contributions of all authors. All authors have given approval to the final version of the manuscript.

## Appendix Supplementary materials

Image, application 1
