# Supplementary material for: Risk analysis to reduce the appearance of antibiotic-resistant microorganisms in drinking water, dust and manure of broiler farms through adjustments of management measures
Source: Poult Sci. 2026 Jan 8;105(3):106411. doi: 10.1016/j.psj.2026.106411 (PMC12860608; doi:10.1016/j.psj.2026.106411)
Supplement: Supplementary file 1 [file mmc1.pdf]

Survey

1. Contact

The data collected is evaluated anonymously. However, if you agree to send us animal feed and manure samples for analysis or to have an inspection and sampling carried out on your farm, you can provide us with your contact details. The provision of the data is on a voluntary basis; for a desired contact, at least one e-mail address must be provided, and the consent below must be given.

<u>Please enter your name:</u>

First name(s):

Surname:

<u>Please enter your address:</u>

Street, house no.:

Postal code:

Location:

Country:

<u>Please indicate how we can contact you:</u>

Telephone number:

E-mail address:

Consent: I agree that TUM may use my data for the following purposes:

- o To send sample bags for independent sampling at the farm.
- o I have already received a package of sample bags. My identification number is as follows:
- o To contact the project organizers regarding a possible farm visit.
- o To participate in the raffle.

- o I hereby confirm the correctness of my data and agree that it may be used by the project organizer to contact me.

2. Personal Data

<u>Please indicate your gender:</u>

- o Male
- o Female
- o Non-binary

- Other:

Please indicate your year of birth (four digits):

Please select your highest (professional) degree:

- no school-leaving certificate
- Primary / secondary school diploma
- Realschule (middle school)
- Upper secondary school (Abitur)
- completed training / journeyman
- Master / Technician
- Businessman
- Study Diploma
- Bachelor's degree
- Master's Degree
- Doctorate
- Other:

3. General Operating Data

Please indicate the number of persons working on your holding (in the poultry sector):

- 1
- 2
- 3
- on 3, namely:

Please select your farm's type of operation:

- Conventional
- Organic
- Other:

What kind of fattened poultry do you keep on your farm?

- I do not keep poultry for fattening on my farm
- Ducks
- Geese
- Broilers
- Turkeys
- Others:

What is the approximate distance to the nearest poultry farm (in km)?

Please enter the distance as a digit:

3.1. Questions concerning staff

<u>What is the employment relationship of these employees?</u>

- No additional members of staff work in the poultry section
- Family member
- Trainee
- Temporary worker
- Permanent staff
- Other:

<u>What is the German language level of your employees?</u>

- fluent, native speaker
- good knowledge of German (spoken and written)
- comprehensible knowledge of German
- poor knowledge of German
- no knowledge of German

4. Fattening Data

<u>Please indicate how long you have been working with your current supplier:</u>

- 0 to 6 months
- 6 to 12 months
- 1 to 2 years
- 2 to 5 years
- 5 to 10 years
- over 10 years

<u>Was the quality of the day-old chickens constant in the last five flocks?</u>

- Yes
- No, it improved.
- No, it decreased.
- No, it fluctuates heavily.

<u>Please select the breed(s) kept on your holding (farm?):</u>

- Cobb, no.:
- Ranger Gold
- Ross, no.:
- Other

<u>Are both sexes fattened?</u>

- Yes
- No, only female
- No, only male.

<u>How many hygienic units (e.g., broiler houses with separate access) are there on your farm?</u>

Please enter the number in digits:

<u>How many animals are fattened per fattening period and hygienic unit (e.g., broiler houses with separate access)?</u>

Please enter the number in digits:

<u>How many fattening periods are carried out on average per year?</u>

Please enter the number in digits:

<u>What is the average fattening time (in days)?</u>

Please enter the duration in digits:

5. Management-Technical Data

<u>Is there an obligation for persons outside the establishment to wear overalls?</u>

- Yes
- No

<u>Is the broiler house accessed via a changing room?</u>

- Yes
- No

<u>Are the broilers delivered on chick paper?</u>

- Yes
- No, but on:

<u>Which flooring/bedding material is used?</u>

- Straw granules
- Softwood chips
- Spelt pellets / granulates
- short chopped straw (max. 3 to 5 mm)
- Maize silage
- Other:

Are there environmental enrichment items available to the animals?

- Yes
- No

Do the animals have access to outdoor areas?

- Yes
- No

Is there a cold scratching area for the animals?

- Yes
- No

Does the broiler house have outdoor climate?

- Yes
- No

5.1. Question on Enrichment Items

What enrichment items are available to the animals?

- Pick stones
- Straw bales
- Separate dust bath areas
- Other:

6. Housing climate

Please indicate the temperature in the broiler house:

Average:

Minimum:

Maximum:

Will the temperature settings remain constant during masting?

- Yes
- No, they decrease with age (from . . . to . . . °C)
- No, they increase with age (from . . . to . . . °C)

Please indicate the relative humidity in the broiler house:

Average:

Minimum:

Maximum:

What type of ventilation is used in the broiler house?

- Vacuum ventilation system
- DC ventilation system
- Overpressure ventilation system
- Other:

Is there a cooling system?

- Yes
- No

6.1. Question about Cooling System and Air Quality

Is the air quality (meaning CO2, ammonia or particulate matter) measured in the broiler house?

- Yes
- No

What type of cooling system is available?

- Roof irrigation
- Spray mist system
- Windchill effect
- Other:

At what intervals is the air quality (meaning CO2, ammonia or particulate matter) measured in the broiler house?

- Air quality is never measured
- Continuously / several times a month
- Monthly
- Every six months
- Annually
- less often

7. Feeding System

Where are the rations planned?

- Completely independent, in-house

- In-house with planning program
- Ration planning by independent consultants
- Ration planning by consultants of a feeding company
- Ration planning by consultants of the quality promotion program/producer association
- Specified by Quality Promotion Program / Producer Community
- Other:

<u>Are additives added to the feed with the intention of promoting intestinal health?</u>

- Yes
- No
- Unknown

<u>Do you feed in phases?</u>

- Yes
- No

7.1 Question about Phase Feeding

<u>Which feed additives are administered for the purpose of promoting intestinal health?</u>

- Please provide a more precise indication of the feed additive used (as given in brackets):
- Macronutrients (e. g. proteins, carbohydrates, fats):
- Micronutrients (e. g. amino acids, vitamins):
- Minerals (e. g. quantitative or trace elements):
- other feed additives (e. g. coccidiostats, enzymes, prebiotics, probiotics, organic acids, essential oils):

<u>How many phases are there and how are they divided?</u>

- Please indicate the duration of each phase from the first to the last day (e. g. phase 1: 1 - 21)
- Phase 1
- Phase 2
- Phase 3
- Phase 4

8. Health Information

<u>Please indicate the therapy frequency of the respective dates:</u>

of the last six months:

of the penultimate six months:

<u>Are precautionary measures taken to minimize the use of antibiotics?</u>

- Yes
- No

When are antibiotics used?

- No antibiotics are used
- In exceptional cases
- Irregular, with the following symptoms:
- Regularly, in the following age (in days):

Are active substances administered via water?

- Yes
- No

8.1. Questions on Health Data

What precautionary measures are being taken?

- Feed additives
- Vaccination programs
- Monitoring of feed consumption
- Monitoring of water expenditure
- Other:

Which active ingredients are regularly used during fattening?

- Aminoglycosides (e. g. Lincospexin powder, Neo-Mix, pyanoside, refobacin)
- Beta-lactams (e. g. amoxicillin, aviapen, hostamox, paracillin, penicillin)
- Fluoroquinolones (e. g. Advocine Pouldre Orale, Baytril, Enrox)
- Lincosamides (e. g. Lincoplexin powder, pyanoside)
- Polypeptides (e. g. Colivet)
- Sulfonamides (e. g. cotrimoxazole, coccidiol, methoxasol-T, sulphaclozin Na, sulphadimidine Na)
- Tetracyclines (e. g. Beladox, Pulodox, Soludox, Tetracycline hydrochloride, TCT-HCl, Ursocycline powder)
- Other:

How else are active substances administered?

- No active substances are used
- There is a single animal treatment
- Other:

Please indicate how long the water pipes are flushed after an active substance administration (in minutes):

9. Cleaning System

Who cleans the broiler house after the fattening?

- o In-house cleaning
- o Cleaning by external company
- o Other:

How long does the broiler house remain empty between removal of the animals and a new stabling (in days)?

Please enter the days as digits:

What products are used for cleaning?

Please also specify the product name:

- o Alkaline barn cleaner
- o Acid barn cleaner
- o Other:

What products are used for disinfection?

Please also specify the product name:

- o Iodine-containing disinfectant:
- o Disinfectant containing peroxide:
- o Acidic disinfectant:
- o Other:

10. Final Page

Thank you for helping the MiniAbeR project by taking part in this survey!

The project is part of the Model and Demonstration Projects (MuD) Animal Welfare of the Federal Livestock Programme. The funding is provided by the Federal Ministry of Food and Agriculture (BMEL) on the basis of a decision of the German Bundestag, project promoter is the Federal Institute for Agriculture and Food (BLE), grant code 2829MDT220.
